# Ameloblastoma demographic, clinical and treatment study - analysis of 40 cases

**DOI:** 10.1590/S1808-86942012000300008

**Published:** 2015-10-14

**Authors:** Luciano José de Lemos França, Otávio Alberto Curioni, Diógenes Lopes Paiva, Débora Modelli Vianna, Rogério Aparecido Dedivitis, Abrão Rapoport

**Affiliations:** a Resident Physician in Head and Neck Surgery and Otorhinolaryngology – Heliópolis Hospital, São Paulo/SP, Brazil.; b PhD in Pathology – Medical School of the University of São Paulo (Head of the Department of Otorhinolaryngology and Head and Neck Surgery - Hospital Heliópolis, São Paulo/SP, Brazil).; c Resident Physician in Head and Neck Surgery and Otorhinolaryngology – Heliópolis Hospital, São Paulo/SP, Brazil.; d Resident Physician in Head and Neck Surgery and Otorhinolaryngology – Heliópolis Hospital, São Paulo/SP, Brazil.; e MD. Professor; Senior Associate Professor - Fundação Lusíada UNILUS.; f Senior Associate Professor – Department of Surgery – Medical School of the University of São Paulo. Technical Director of the Hospital Heliópolis, São Paulo/SP, Brazil).

**Keywords:** ameloblastoma, mandibular neoplasms, maxillary neoplasms, odontogenic tumors

## Abstract

Dental lesions represent about 1% of oral cavity tumors being ameloblastoma the most common one. It is a tumor of epithelial origin that mainly affects the jaw, and less commonly the maxilla. Its clinical presentation is that of an asymptomatic slow-growing tumor. Despite being a benign tumor, it has an invasive behavior with a high rate of recurrence if not treated properly.

**Objective:**

To describe the cases of ameloblastoma in a reference department.

**Methods:**

Retrospective analysis of 40 cases. The variables analyzed were: age, gender, ethnicity, tumor location, type of treatment, complications and recurrence.

**Results:**

The most affected gender was male - 21 cases (52.5%); with a predominance of Caucasians - 24 cases (60%). The mean age was 35.45 years; the most common location was in the jaw - 37 cases (92.5%). Facial asymmetry was the most frequent complaint. Of the 40 cases, 33 were submitted to surgery. Of those submitted to surgery, 24 (72.72%) underwent segmental resection, with recurrence in 4 (12.12%) cases.

**Conclusion:**

Ameloblastoma may relapse when treatment is not performed with broad surgical resection of the lesion with wide safety margins.

## INTRODUCTION

Ameloblastoma is a benign neoplasia which can be locally invasive. Among odontogenic neoplasia, the ameloblastoma affects the bones of the maxillomandibular complex, representing the odontogenic tumor of higher clinical significance^1^. The term ameloblastoma was first utilized in 1930, when an odontogenic tumor was described with multiple cords and interconnected cell laminas, of epithelial origin and homologous with the dentogingival lamina of onset during odontogenesis^2^. It may originate from remains of the dental lamina, reduced epithelium from the enamel, epithelial remains of Malassez or from the basal cells of the surface epithelium^3^, ^4^.

The classification of this neoplasia, by the World Health Organization (WHO), places it as a tumor stemming from the odontogenic epithelium without ectomesenchima^5^.

The ameloblastoma has a relatively low incidence, encompassing only 1% of all maxilla and mandible tumors, most of the cases being diagnosed between the third and fifth decades of life^6^. It is a slow growth tumor, with only few symptoms in initial stages^7^. Despite being benign, it has an invasive behavior with a high rate of recurrence if not treated properly^8^.

The goal of the present paper is to do a descriptive review of ameloblastoma cases admitted in a reference head and neck cancer treatment center.

## PATIENTS AND METHODS

The present study was approved by the Ethics in Research Committee of the Institution where it was carried out.

We made a retrospective analysis of 40 charts, from patients diagnosed with ameloblastoma, between 1978 and 2011. We assessed clinic-demographic and histopathological characteristics of these cases. We analyzed the following variables: age, gender, ethnicity, tumor location, type of treatment, complications and recurrence. This study had a descriptive statistical analysis.

## RESULTS

In this series, there were 21 (52.5%) cases that were males and 19 (47.5%) females. Twenty-four (60%) cases were Caucasians and 16 (40%) were non-Caucasians. The mean age was 35.45 years (ranging between 14 and 65).

**Figure 1:**
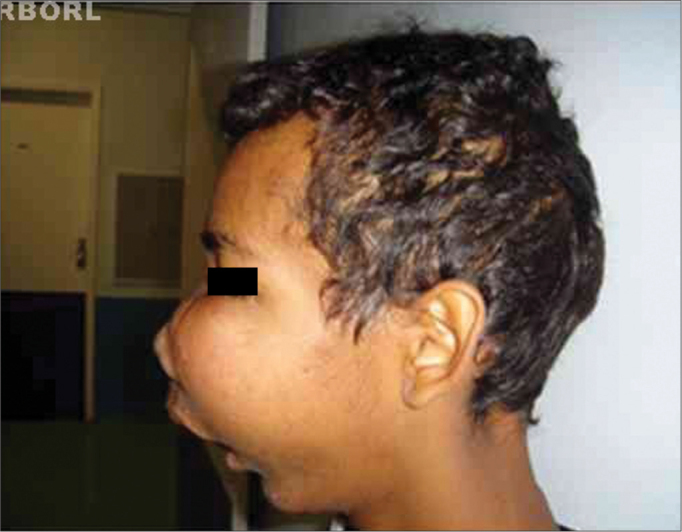
Recurrence of maxilla ameloblastoma.

The mandible was the most affected site. Nineteen (47.5%) patients had tumors restricted to the mandible body. Of these, five (12.5%) went beyond the mid-line. Eight (20%) had involvement of the mandible body and angle or angle and ramus; in 10 (25%) cases the lesion advanced to the body, angle, ramus and condyle; and three cases (7.5%) involved the maxilla - a less frequent location (Figure 1).

Facial asymmetry was the foremost complaint in 65% (26 cases). Thirty-three (82.5%) patients were submitted to a surgical procedure. Of these, 24 (72.72%) were submitted to segment resection of the mandible; five (15.15%) to enucleation or curettage, one (3.03%) to marginal resection of the mandible and three (9.09%) to maxillectomy. The mandibular reconstruction, when indicated, was carried out with the miniplate for mandibular reconstruction and/or iliac crest bone graft.

**Figure 2:**
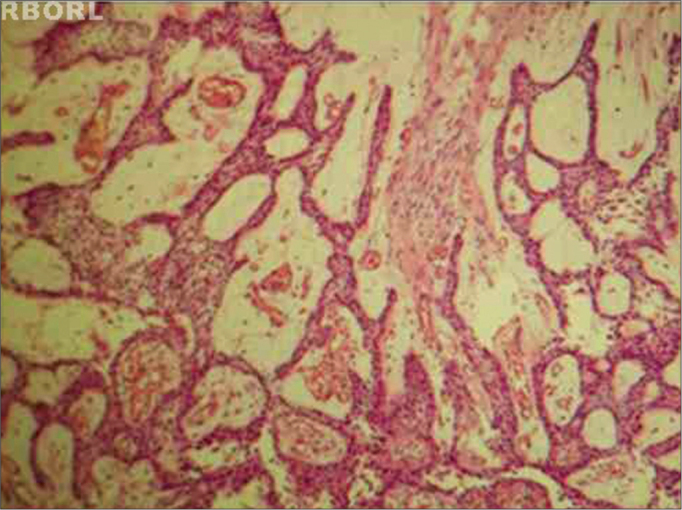
Plexiform pattern ameloblastoma, showing the cystic-type degeneration. HE 100x.

As far as surgical complications are concerned, there were wound infections in eight (24.24%) patients. Tumor recurrence in four cases (12.12%), three (9.09%) after curettage and in one (3.03%) it happened after maxillectomy. Of the three (9.09%) patients who had a recurrence after curettage, two (6.06) were plexiform and one (3.03%), unicystic (Figure 2).

## DISCUSSION

Gender distribution was pretty much the same, 52.5% men and 47.5% women, which is agreement with data described in the literature^9^. In our study, there was a predominance of Caucasians - 60% of the cases.

The diagnosis of ameloblastoma can be obtained by means of a panoramic x-rays done in dental care routine, showing intraosseous growth or, in more advanced cases, with expansion of the bone cortical, determining facial asymmetry - which was the most common complaint in our series. We stress that most of these cases had already been treated.

As far as location is concerned, the mandible was the most frequent site, with 92.5%; three (7.5%) patients had a maxilla tumor. The incidence of ameloblastoma, as to anatomical location, is almost exclusive in the mandible, happening in 80% to 97% of the cases. Its occurrence in the maxilla is rare, varying between 3% and 13%^4^, ^10^. According to the literature, in the maxilla, 47% of the cases are in the posterior region and 15% in the maxillary sinus and nasal cavity floor^11^, ^12^. There is no gender predilection. In our experience, two cases were posterior and one case, treated anteriorly, affected both maxillary sinuses. Ameloblastoma rarity in the maxilla may cause diagnosis and treatment difficulties^13^.

There is no standardization in the literature as to mandible regions affected. Lunardi et al.^8^ suggest division in anatomical areas and not in dental regions, as per described by Reichart et al.^9^.

Since we are a reference center for cancer treatment, and ameloblastoma is a benign tumor, the cases referred were either advanced, with large facial deformities and complex resections, or had been submitted to some type of previous treatment, unsuccessfully.

In our series, 20 (50%) patients had already been submitted to some type of surgery. Ten cases (25%) had extensive lesions which involved the mandible body, angle, ramus and condyle, besides five cases (12.5%) of body involvement which went beyond the midline, and one case (2.5%) of bilateral maxilla involvement, making up a total of 16 (40%) cases of extensive lesions.

There are numerous methods for treatment, including enucleation and/or curettage, marginal en-block resection or hemiressection (hemimaxillectomy or hemimandibulectomy), cryotherapy, radiotherapy, and surgery - the most indicated treatment approach. The choice of treatment depends on lesion size, type, location and general patient condition. In this study, 24 (72.72%) were submitted to segment resection, five (15.15%) cases submitted to enucleation or curettage, one (3.03%) to marginal resection and three (9.09%) to maxillectomy. Surgery is the only treatment for ameloblastoma, because of its resistance to radiotherapy^14^. After segment resection, it is essential to rebuild it, with bone graft and/or a titanium plate and screws^15^.

Insofar as surgical complications are concerned, there was wound infection in eight (24.24%) patients, with the need for antibiotic treatment, of whom two had bone graft loss. Four (12.12%) patients had recurrences, three (9.09%) after curettage and one (3.03%) after maxillectomy.

Treatment of the cases investigated in this study was carried out only after biopsy results indicated ameloblastoma. Not specifying the histological type – and because of that we used the histological reclassification^15^, which is of the utmost importance because of the behavior of the most common types (follicular, plexiform and unicystic) which presented and influence treatment^9^. For the plexiform and follicular types, radical surgery is the best procedure, with a safety margin of 1.5 and 3.0 cm and, for the unicystic type, bone curettage is indicated^16^.

The bilateral maxillectomy recurrence happened to one patient coming from another clinic, with a prior history (five years before) of the maxilla follicular variant of ameloblastoma. In our service, the lesion was 15cm long in its longest axis, occupying the entire facial structure, from the orbit floor all the way to the hard palate, in the craniocaudal direction and from the nasal cavities to the rhinopharynx, in the anteroposterior direction.

## CONCLUSION

The ameloblastoma is usually of late diagnosis because of its poor symptomatology and low prevalence. In our experience, curettage did not prove to be an efficient treatment. Its treatment requires, preferably for advanced tumors, the resection with safety margins and immediate reconstruction whenever possible. There is a need for a routine histological classification of the ameloblastoma for its morphological characterization and, thus, a better treatment definition. Nonetheless, the main success factor associated with the treatment is the early diagnosis and the first efficient and preferable treatment in specialized services.

## References

[1] Neville BW, Damm DD, Allen CM, Bouquot JE (2004). Patologia Oral & Maxilofacial..

[2] Ivey RH, Churchill HR (1930). The need of a standardized surgical and pathological classification of tumors and anomalies of dental origin. Am Assoc Dent Sch Trans..

[3] Hughes CA, Wilson WR, Olding M (1999). Giant ameloblastoma: report of an extreme case and a description of its treatment. Ear Nose Throat J..

[4] Williams TP (1993). Management of ameloblastoma: a changing perspective. J Oral Maxillofac Surg..

[5] Kramer IRH (1992). The World Health Organization: Histological typing of odontogenic tumors: an introduction to the second edition. J Dent Assoc S Afr..

[6] Gorlin RJ, Gorlin RJ, Goldman HM (1970). Thomas' Oral Pathology..

[7] Lucas RB, Lucas RB (1964). Patology of tumors of the oral tissues..

[8] Lunardi LV, Fava AS, Martins RH, Homem MGN, Rapoport A, Carvalho MB (2001). Tratamento cirúrgico do ameloblastoma com reconstrução de mandíbula com enxerto de crista ilíaca não vascularizado – estudo de sete casos. Rev Col Bras Cir..

[9] Reichart PA, Philipsen HP, Sonner S (1995). Ameloblastoma: Biological profile of 3677 cases. Eur J Cancer B Oral Oncol..

[10] Gold L (1991). Biologic behavior of ameloblastoma. Oral Maxillofac Surg Clin North Am..

[11] Iordanidis S, Makos C, Dimitrakopoulos J, Kariki H (1999). Ameloblastoma of the maxilla. Case report. Aust Dent J..

[12] Nastri AL, Wiesenfeld D, Radden BG, Eveson J, Scully C (1995). Maxillary ameloblastoma: a retrospective study of 13 cases. Br J Oral Maxillofac Surg..

[13] Chedid HM, Amar A, Rapoport A, Cardoso R, Curioni OA (2011). Ameloblastoma de maxila: Estudo de 3 casos. Rev Bras Cir Cabeça Pescoço..

[14] Li KK, Fabian RL, Goodman ML (1997). Malignant fibrous histiocytoma after radiation for ameloblastoma of the maxilla. J Oral Maxillofac Surg..

[15] Vasan NT (1995). Recurrent ameloblastoma in an autogenous bone graft after 28 years: a case report. N Z Dent J..

[16] Martins RH, Andrade Sobrinho J, Rapoport A, Rosa MP (1999). Histopathologic features and management of ameloblastoma: study of 20 cases. São Paulo Med J..

